# Thermal Conductivity of Magnesium Oxide From Absolute, Steady-State Measurements

**DOI:** 10.6028/jres.103.021

**Published:** 1998-08-01

**Authors:** A. J. Slifka, B. J. Filla, J. M. Phelps

**Affiliations:** National Institute of Standards and Technology, Boulder, CO 80303

**Keywords:** guarded hot plate, magnesium oxide, thermal conductivity

## Abstract

The thermal conductivity of polycrystalline magnesium oxide has been measured over the temperature range from 400 K to 1300 K using a modified guarded-hot-plate design. Three different thicknesses of specimens having 93 % of theoretical density were tested to verify the operation, accuracy, and reproducibility of our apparatus. The measured thermal conductivity ranges from 30 W · m^−1^ · K^−1^ down to 8 W · m^−1^ · K^−1^ and has an inverse-temperature functionality. The results agree well with literature values for this material.

## 1. Introduction

We have measured the thermal conductivity of magnesium oxide using an absolute, steady-state technique. We chose magnesium oxide because the thermal conductivity of this dense, polycrystalline material has been measured often enough for the thermal behavior to be considered well-known [1]. Our experimental technique, a one-sided guarded hot plate, is described in detail elsewhere [2]. Measurement of the thermal conductivity of magnesium oxide serves two purposes: to validate the design and operation of our apparatus and to measure a well-behaved material with relatively high thermal conductivity. We will measure monolithic ceramics as well as thermal barrier coatings with this apparatus and technique. The coated specimens use substrates of stainless steel or nickel-based superalloys. These substrate materials have the same magnitude of thermal conductivity as magnesium oxide, so these tests give us an idea of how accurate we can expect our thermal conductivity measurements of substrate materials to be. This range of thermal conductivity is on the high end of the design measurement range of the apparatus. We explain the validation of the design and operation in the experimental procedure section of the paper.

Comparative data for the thermal conductivity of magnesium oxide is readily available [1,3,4]. We compare our results to recommended values from the literature [3].

## 2. Experimental Procedure

**Fig. 1:**
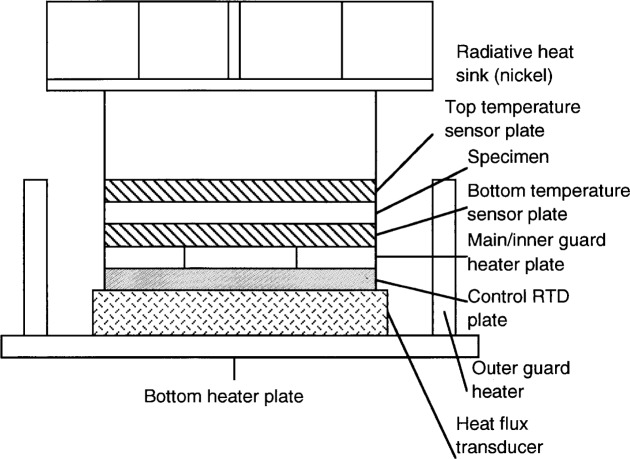
Schematic drawing of the measurement stack of the guarded hot plate.

The tests were done using a one-sided guarded hot plate, which is a modified version of the ASTM C 177 specification [5]. The conductivity was measured over a temperature range from 400 K to 1300 K. Figure 1 shows a schematic drawing of the salient features of the apparatus. The specimen rests between two sensor plates and experiences an upward, one-dimensional heat flow because the lower part of the measurement stack is an isothermal hot “cup.” Since the apparatus operates at high temperature, up to 1300 K, a thermal grease cannot be used between the specimen and sensor plates. A pliable metal like indium cannot be used due to its low melting temperature, and metals that can handle the high temperature are too stiff to give intimate thermal contact over the large surface area of the 69.75 mm diameter specimen. Additionally, a metal foil to provide intimate thermal contact between specimen and sensor plates is not feasible because type-s thermocouples embedded in the surface of the sensor plates would short if a metal foil were used. We are left having to measure the thermal resistance between our specimen and sensor plates. The Fourier conduction equation in one dimension modified to include this additional thermal resistance term is

$$\frac{\Delta T\cdot A}{Q}=\frac{\Delta x}{k}+2\cdot R_{T},$$ (1)

where Δ*T* is the temperature difference, *A* is the cross-sectional area that heat flows through, *Q* is the heat flow rate, Δ*x* is the length over which the temperature difference is measured, *k* is the thermal conductivity, and *R*_T_ is the specific interfacial thermal resistance. By measuring two specimens of different thickness, we can solve Eq. (1) for the two unknowns *k* and *R*_T_. Therefore we generate a set of thermal conductivity results for each combination of two specimen thicknesses tested. An uncertainty analysis of our system gives a 5 % relative standard uncertainty for our experiments [2, 6].

Since there are no high-temperature ceramic standards of thermal conductivity in the United States, we wanted to use a material with a large measurement history to critically evaluate our apparatus and experimental procedure. Magnesium oxide has an accepted thermal conductivity, and therefore we can evaluate our method of using two specimens of differing thickness to simultaneously measure thermal conductivity of the specimen and specific thermal contact resistance between the specimen and sensor plates.

## 3. Results

The specimens are commercial, sintered disks of polycrystalline magnesium oxide. They are 69.75 mm in diameter and have thicknesses of 2.59 mm, 5.04 mm, and 7.64 mm. The surface finish of the specimens varies from specimen to specimen and ranges from 0.2 μm to 0.5 μm centerline average roughness. This variation in surface finish is relatively small, and, in our experience, should provide a consistent thermal contact resistance from specimen to specimen. The specimens have 93 % of the theoretical density, based on measurements of mass and dimensions, using 3.581 g · cm^−3^ as the theoretical density of magnesium oxide [7]. The average grain size is 25 μm, measured by optical microscopy of a fracture surface.

**Fig. 2:**
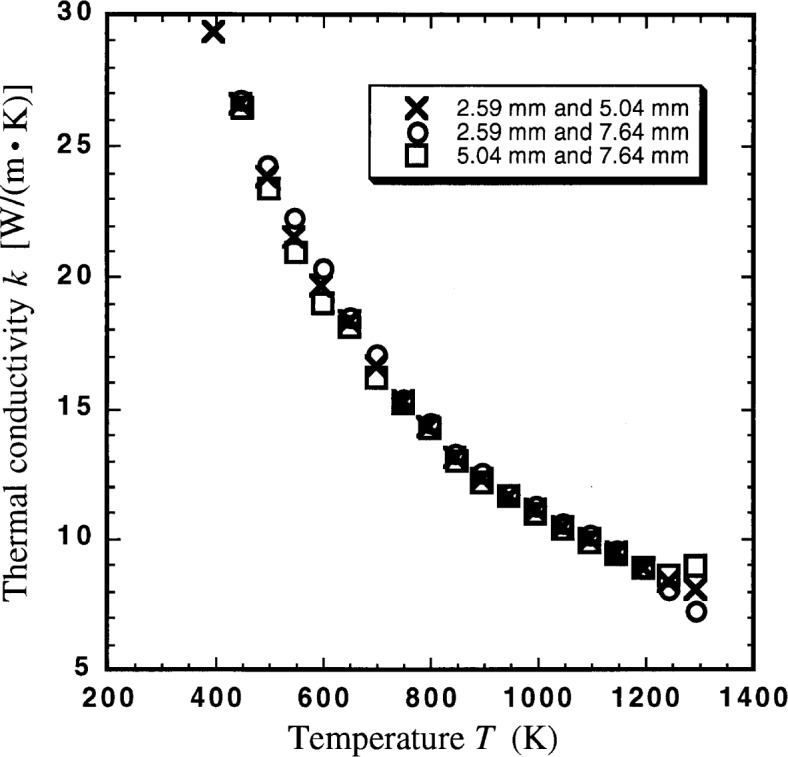
Thermal conductivity *k* for the three pairings of the three thicknesses of magnesium oxide specimens tested.

**Fig. 3:**
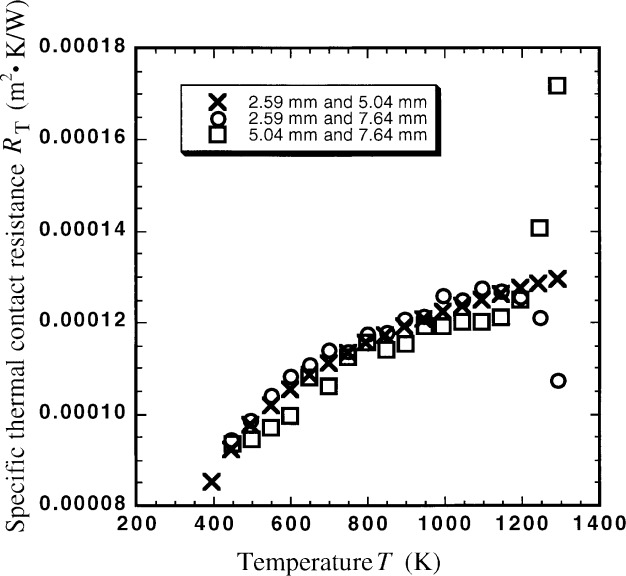
Specific thermal contact resistance *R*_T_ for the three pairings of the three different thicknesses of magnesium oxide specimens tested.

We measured three different thicknesses of specimens in order to determine whether our method for simultaneously determining thermal conductivity *k* and specific interfacial thermal resistance *R*_T_ would be valid. The data from three experiments were paired three different ways and Eq. (1) was solved. We expected three curves for thermal conductivity, all within the measurement uncertainty of one another if the experimental and analytical methods were valid. Figure 2 shows the thermal conductivity results for the three specimen pair combinations. Table 1 shows the thermal conductivity data for the three pairs of data from the three thicknesses of specimens tested. A measurement of the 7.64 mm thick specimen was not made at 397.8 K, thus there are two blanks in Table 1. The values of *k* range from 30 W · m^−1^ · K^−1^ down to 8 W · m^−1^ · K^−1^ as temperature increases and are consistent with each other to within 0.05 *k*, except for the data at 1300 K. Failures at 1300 K in the platinum wiring for two of the three data sets may account for the additional spread at that temperature. The spread in the data at 1300 K was less than 0.1 *k*, so these data were left in for completeness. Figure 3 shows the specific thermal contact resistance *R*_T_ between the specimen and a sensor plate for the three combinations of the three different specimen thicknesses. The data shown are for one interface, so the total specific thermal contact resistance would be two times that shown in the figure. The experimental condition must be met that the thermal resistance due to the specimen be at least 4 times that due to the interface [2]. The relative standard uncertainty in the measurement of thermal conductivity is 5 % for the NIST guarded hot plate, thus the relative uncertainty in the interfacial resistances *R*_T_ is 20 % [2]. The specific thermal contact resistances are consistent within 0.1 *R*_T_, except for the two outliers at 1300 K, which are probably due to the sensor failures at 1300 K. The magnitude and the functionality of the specific thermal contact resistance data are similar to those from measurements we have made on ceramic glass and partially-stabilized zirconia monolithic specimens.

**Fig. 4:**
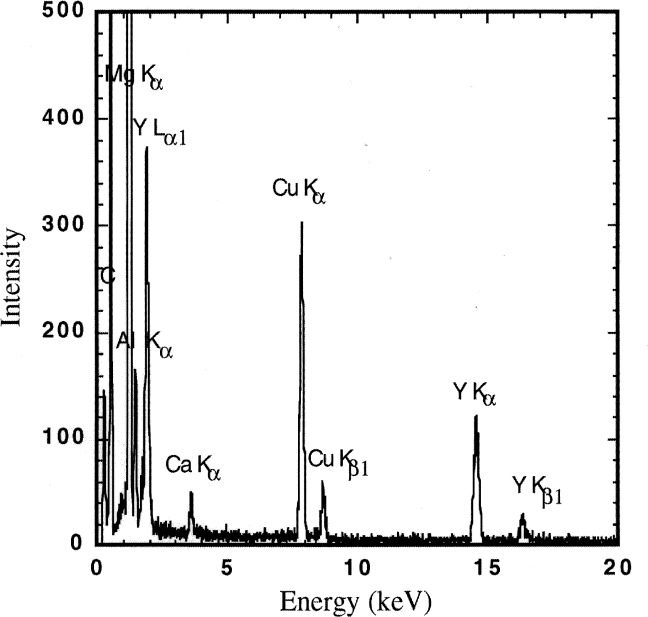
Analytical electron microscope (AEM) analysis of a spot in the magnesium oxide sample that shows representative impurities.

**Fig. 5:**
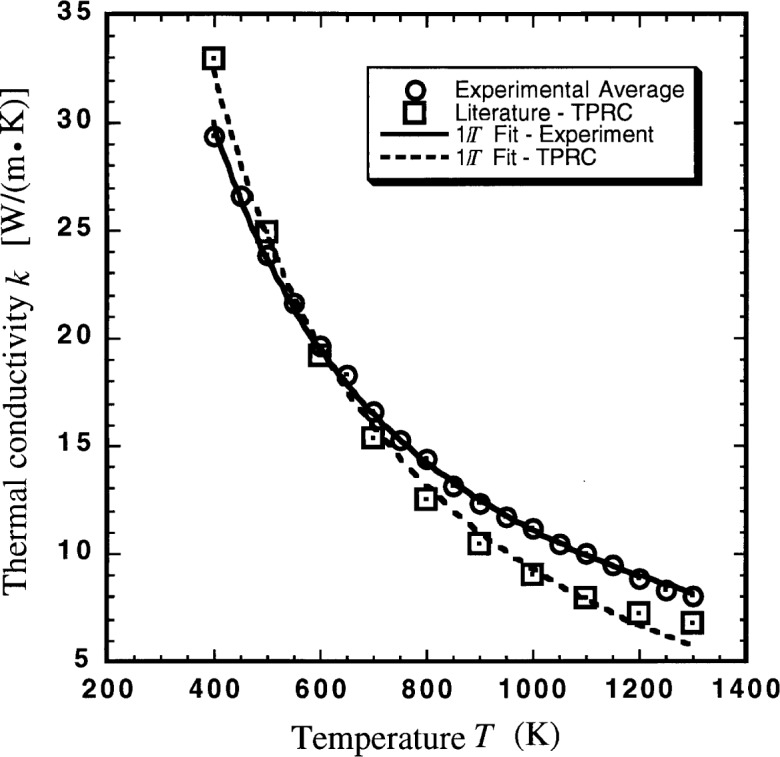
Comparison of thermal conductivity results for our average experimental data and literature values [3], including inverse-temperature curve fits for each.

The thermal conductivity data obtained from these experiments compare well with data found in the literature [1,3,4]. Small variations can occur due to different grain sizes in polycrystalline material, and especially from impurities. The purity of a representative specimen was checked using energy dispersive spectrometry (EDS) with both scanning electron microscope (SEM) and analytical electron microscope (AEM) samples. Analysis with the SEM showed mostly strong magnesium peaks. Some spots were found, though, that showed aluminum, yttrium, and calcium impurities. Characteristic x-ray maps showed that the impurities are distributed randomly throughout the sample. AEM analysis showed similar results but provides higher resolution of peaks. Figure 4 shows the AEM spectra of a spot showing impurities. The copper and carbon peaks are from the carbon-coated copper grid used to hold the ground sample in the AEM. Based on crude analysis of the peaks and the number of spots found to have impurities versus the number found without impurities, we estimate the total mass fraction of impurities to be around 1 %. Even an amount this small can alter the thermal behavior of a material such as magnesium oxide that has a simple crystalline structure [8]. Analysis of all of the thermal conductivity data from these experiments results in a best-fit line among simple functions of an inverse-temperature functionality. Figure 5 shows a comparison of our fitted data and an inverse-temperature fit to recommended literature values for magnesium oxide [3], corrected to a density of 93 % of the theoretical value using the relation [4]:

$$k_{\text{porous}}=\frac{k_{\text{dense}}}{\left(1–P\right)},$$ (2)

where *k*_porous_ is the thermal conductivity of a porous specimen, *P* is the porosity of the specimen and *k*_dense_ is the thermal conductivity of dense material. The experimental results obtained here agree with the recommended literature values within the combined stated uncertainty of the literature values and the standard uncertainty of these experiments. Table 2 shows the average experimental values and the literature values [3]. The literature values are quoted at the 100 K interval given in Touloukian [3]. The temperature dependence of our results appears to be slightly different from the recommended literature values.

According to the theory of thermal conductivity of solids, conductivity should show an inverse-temperature functionality above the Debye temperature of the material [4, 7]. The Debye temperature of magnesium oxide is about 750 K [9]. Separately fitting our average experimental data and the recommended literature values from 750 K to 1300 K using an inverse-temperature function statistically shows that our data fit slightly better than the recommended literature values. The standard uncertainty of the inverse-temperature fit to the literature values is 0.12, whereas the average experimental results have a standard uncertainty for the same function of 0.06 [6]. As the fit is extended down to lower and lower temperatures, our experimental data still fit the inverse-temperature function well, whereas the recommended literature values fit this function with increasing uncertainty.

## 4. Conclusions

We have measured the thermal conductivity of polycrystalline magnesium oxide using an absolute, steady-state technique. The conductivity was measured over a temperature range of 400 K to 1300 K. As far as we know, this is the only absolute, steady-state measurement of thermal conductivity over this temperature range.

Three different thicknesses of specimen were used so that we could analyze the data in three different pairings to solve for the two unknowns in Eq. (1), thermal conductivity *k* and specific thermal contact resistance *R*_T_. The results verify the operation and accuracy of our apparatus and our data-analysis method, as the data are consistent within the standard uncertainty of the measurement. The results compare well with accepted literature values, and based on theoretical considerations [4, 8], are at least as good as the recommended literature values for polycrystalline magnesium oxide.

## Figures and Tables

**Table 1:** Experimental values of thermal conductivity *k* for the three pairs of data from the three thicknesses of magnesium oxide specimens indicated in square brackets

*T*	*k*	*k*	*k*
Temperature	[2.59 mm and 5.04 mm]	[2.59 mm and 7.64 mm]	[5.04 mm and 7.64 mm]
(K)	(W · m^−1^ · K^−1^)	(W · m^−1^ · K^−1^)	(W · m^−1^ · K^−1^)
397.8	29.4		
447.8	26.6	26.8	26.4
497.8	23.8	24.3	23.4
548.1	21.6	22.3	20.9
598.4	19.6	20.4	19.0
648.5	18.3	18.5	18.1
698.5	16.6	17.1	16.1
748.4	15.3	15.4	15.2
798.1	14.3	14.4	14.3
847.6	13.1	13.2	13.0
897.3	12.3	12.5	12.2
947.3	11.7	11.7	11.6
997.2	11.1	11.3	11.0
1046.9	10.5	10.6	10.4
1096.6	10.0	10.1	9.8
1146.2	9.5	9.6	9.4
1195.4	8.9	8.9	8.9
1243.7	8.3	8.0	8.6
1292.4	8.0	7.3	8.9

**Table 2:** Average experimental and comparative literature values for thermal conductivity *k* of magnesium oxide having a density of 93 % of the theoretical value 3.581g · cm^−3^ [3]

Temperature	Experimental, average	Literature
(K)	(W · m^−1^ · K^−1^)	(W · m^−1^ · K^−1^)
400	29.4	33.0
450	26.6	
500	23.8	24.9
550	21.6	
600	19.7	19.2
650	18.3	
700	16.6	15.3
750	15.3	
800	14.3	12.5
850	13.1	
900	12.3	10.4
950	11.7	
1000	11.1	9.1
1050	10.5	
1100	10.0	7.9
1150	9.5	
1200	8.9	7.2
1250	8.3	
1300	8.1	6.8
